# Reduction of microwave ablation needle related metallic artifacts using virtual monoenergetic images from dual-layer detector spectral CT in a rabbit model with VX2 tumor

**DOI:** 10.1038/s41598-021-88853-w

**Published:** 2021-04-29

**Authors:** Guorong Wang, Qinzong Gao, Zhiwei Wang, Xiaomei Lu, Shenghui Yu, Zhengyu Jin

**Affiliations:** 1 Department of Radiology, Peking Union Medical College Hospital, Peking Union Medical College, Chinese Academy of Medical Sciences, No.1 Shuaifuyuan Wangfujing Dongcheng District, Beijing, 100730 China; 2 CT Clinical Science, Philips Healthcare, Shenyang, 110016 China; 3 CT Clinical Science, Philips Healthcare, Beijing, 100600 China

**Keywords:** Cancer, Cancer imaging, Cancer screening

## Abstract

The purpose of the study was to investigate the application of virtual monoenergetic images (VMIs) in reducing metal artifacts in rabbit VX2 liver cancer models treated with microwave ablation (MWA) therapy. A total of 31 VX2 liver cancer models that accepted CT-guided percutaneous microwave ablation were analyzed. Conventional images (CIs) with the most severe metallic artifacts and their corresponding energy levels from 40 to 200 keV with 10 keV increment of VMIs were reconstructed for further analysis. Objective image analysis was assessed by recording the attenuation (HU) and standard deviation of the most severe hyper/hypodense artifacts as well as artifact-impaired liver parenchyma tissue. Two radiologists visually evaluated the extent of artifact reduction, assessed data obtained by a diagnostic evaluation of liver tissues, and appraised the appearance of new artifacts according to the grade score. Statistical analysis was performed to compare the difference between CIs and each energy level of VMIs. For subjective assessment, reductions in hyperdense and hypodense artifacts were observed at 170–200 keV and 160–200 keV, respectively. The outcomes of the diagnostic evaluation of adjacent liver tissue were statistically higher at 140–200 keV for VMIs than for CIs. In terms of objective evaluation results, VMIs at 90–200 keV reduced the corrected attenuation of hyperdense and of artifact-impaired liver parenchyma compared with CIs (P < 0.001). When VMIs at 80–200 keV decreased the hypodense artifacts (P < 0.001). Therefore, we concluded that VMIs at 170–200 keV can obviously decrease the microwave ablation needle-related metal artifacts objectively and subjectively in rabbit VX2 liver cancer models.

## Introduction

Microwave ablation (MWA) is a technique that is effective in destroying tumor tissue owing to its dielectric heating mechanism^1,2^. MWA has been widely used to treat liver cancer, and the rabbit VX2 liver tumor model is the most commonly used animal model for percutaneous MWA procedures^3^. Computed tomography (CT) is considered to be one of the typical image-guidance techniques for MWA therapy^4^. The inserted location of the microwave antenna within the targeted tumor influences the efficacy of MWA. It is known that metallic microwave antennas can generate artifacts because of beam hardening, photon starvation, and increased scattering and noise^5^. Thus, the metallic ablation antenna used in interventional procedures can create artifacts on conventional images (CIs), especially images of animals. Whether the ablation process is accurate or effective greatly depends on the relationship between the microwave ablation antenna position and the lesion. Nevertheless, pronounced artifacts around the metallic microwave ablation needle are not suitable to use to determine the association between the microwave ablation antenna position and the lesion and can even cover the display of the target lesion. Moreover, MWA will further influence the adjustment of the puncture position of the ablation antenna. Therefore, reducing metallic material-related artifacts is vitally important for MWA treatment.

There are various methods to reduce metal artifacts obtained from CIs. The application of high tube voltage and increased tube current, appropriate reconstruction procedures, a narrow collimation and larger layer thickness is recommended to achieve metal artifact reduction (MAR)^6^. However, subjects experience an additional dose of radiation due to increased tube voltage and tube current^7^. Hence, an appropriate image postprocessing method needs to be developed for clinical practice.

Virtual monoenergetic images (VMIs) generated from dual-layer detector spectral CT (DLSCT) are a helpful means for MAR. DLSCT can separate high- and low-energy photons^8^, which makes it possible to reconstruct VMIs with different energy levels. VMIs with higher keV values have higher beam hardening resistance. Some studies have shown that this reconstruction technology can reduce metal artifacts created by human metallic implants^9–12^, but it has rarely been applied to MWA therapy for liver cancer. The purpose of this animal study was to investigate the application of VMI in reducing metal artifacts in liver cancer in a rabbit VX2 model treated with MWA.

## Methods and materials

The study was carried out in compliance with the ARRIVE guidelines.

### Establishment of rabbit VX2 model

This study was approved by the Ethics Committee of Peking Union Medical College Hospital and performed in accordance with the National Institutes of Health for the care and use of laboratory animals. The animals used as their own control in the present study because it is designed to test the feasibility of a reconstruction method. We chose a small sample size because the objective of the preclinical study was mainly to assess the application of VMIs in reducing metal artifacts during microwave ablation process. Thirty-one New Zealand white rabbits (male or female, 3.32 ± 0.19 kg) were purchased from our study institution. The individual rabbit was seen as the experimental unit in this study. Approximately 1 mm^3^ of VX2 carcinoma tissue was injected into the liver parenchyma of rabbits by CT-guided percutaneous puncture. The rabbits with tumors were injected intramuscularly with a dose of 40,000 units of penicillin to prevent infection. Randomization was not used to allocate experimental units, since the images of rabbits that been reconstructed at different energy levels were considered as their own control in this study.

### CT-guided percutaneous MWA process

The marked skin surface site was sterilized and draped in a sterile fashion under general anesthesia (3% pentobarbital sodium injected intravenously through the ear vein, 1 ml/kg). All ablations were performed by a microwave ablation system (2450 MHz generator, MICRO TECH, KANG YOU, Nanjing, China) and a 19-gauge antenna. The antenna was inserted into the tumor lesion with the guidance of DLSCT (IQon spectral CT system, Philips Healthcare, Cleveland, USA). The acquisition parameters were as follows: tube voltage 120 kVp, tube current 100 mAs, collimation 64 × 0.625 mm, pitch 1.171 and gantry rotation time 0.75 s, slice thickness 3 mm, and slice interval 3 mm. All the MWA procedures were performed at a constant power of 40 W, and the ablation time per tumor was 1 min. The ablation margin of the tumor was at least 5 mm. The average size of lesions was 0.92 ± 0.22 mm.

The rabbits were included within this study if they accepted successful MWA treatment. The rabbits were excluded if they died prematurely due to any reasons. All operations on rabbits were performed by the same doctor.

The Philips ISP (IntelliSpace Portal version 6.5) workstation was used to conduct standard reconstruction and spectral-based image (SBI) reconstruction on all scanned images. The reconstruction slice thickness and slice interval were both 1 mm. Conventional mixed energy CT image reconstruction was performed by following the hybrid iterative reconstruction method (iDose^4^ level 3). The single VMI was obtained from the SBI dataset (40 keV to 200 keV with 10 keV increments). The slice thickness and slice section increment of the single VMI were the same as those of CI. The window width and window level were 350 HU and 60 HU, respectively.

The volume CT dose index (CTDI_vol_) of each rabbit was recorded.

### Evaluation methods

Two radiologists (with 4 and 11 years of experience in abdominal imaging) reviewed the CI and each level of VMI through the Philips ISP (IntelliSpace Portal version 6.5) workstation with blind independent reading. The images with the most pronounced artifacts, including microwave ablation needles and tumor lesions, were selected for further analysis. Disagreements were resolved through the mutual discussion and negotiation between the two radiologists based on their respective radiology experience in radiology.

### Subjective image analysis

A total of 17 single levels of VMIs were selected for qualitative analysis. The two radiologists mentioned earlier independently assessed the extent of hyper- and hypodense artifacts and the diagnostic quality of surrounding liver parenchyma by 5-point Likert scales; they further evaluated whether VMIs demonstrated new artifacts compared to CI according to 3-point Likert scales. The subjective assessment of the image quality is based on the following aspects^10,12^:

(i) extent of hyperdense (artifacts with higher CT attenuation) and hypodense artifacts (artifacts with lower CT attenuation): 5-metal artifacts are/almost absent; 4-minor artifacts; 3-moderate artifacts; 2-pronounced artifacts; 1-massive artifacts);
(ii) diagnostic assessment of surrounding liver parenchyma around artifacts: 5-full diagnostic quality without artifacts, the surrounding liver parenchyma showing pretty clearly; 4-minor artifacts do not influence diagnostic interpretability, and the surrounding liver parenchyma showing clearly; 3-diagnostic interpretability is only marginally influenced by minor artifacts, and the surrounding liver parenchyma showing moderate clearly; 2-restricted diagnostic interpretability, and the surrounding liver parenchyma showing restrictively; 1-insufficient diagnostic interpretability with the surrounding liver parenchyma covered by metallic artifacts;
(iii) appearance of new/unexpected artifacts compared to CIs: 3-no signs of new/unexpected artifacts are observed; 2-new/unexpected artifacts appear but without impairing the diagnostic assessment; 1-new/unexpected artifacts appear with impairing the diagnostic assessment.

### Objective image analysis

**Figure 1:**
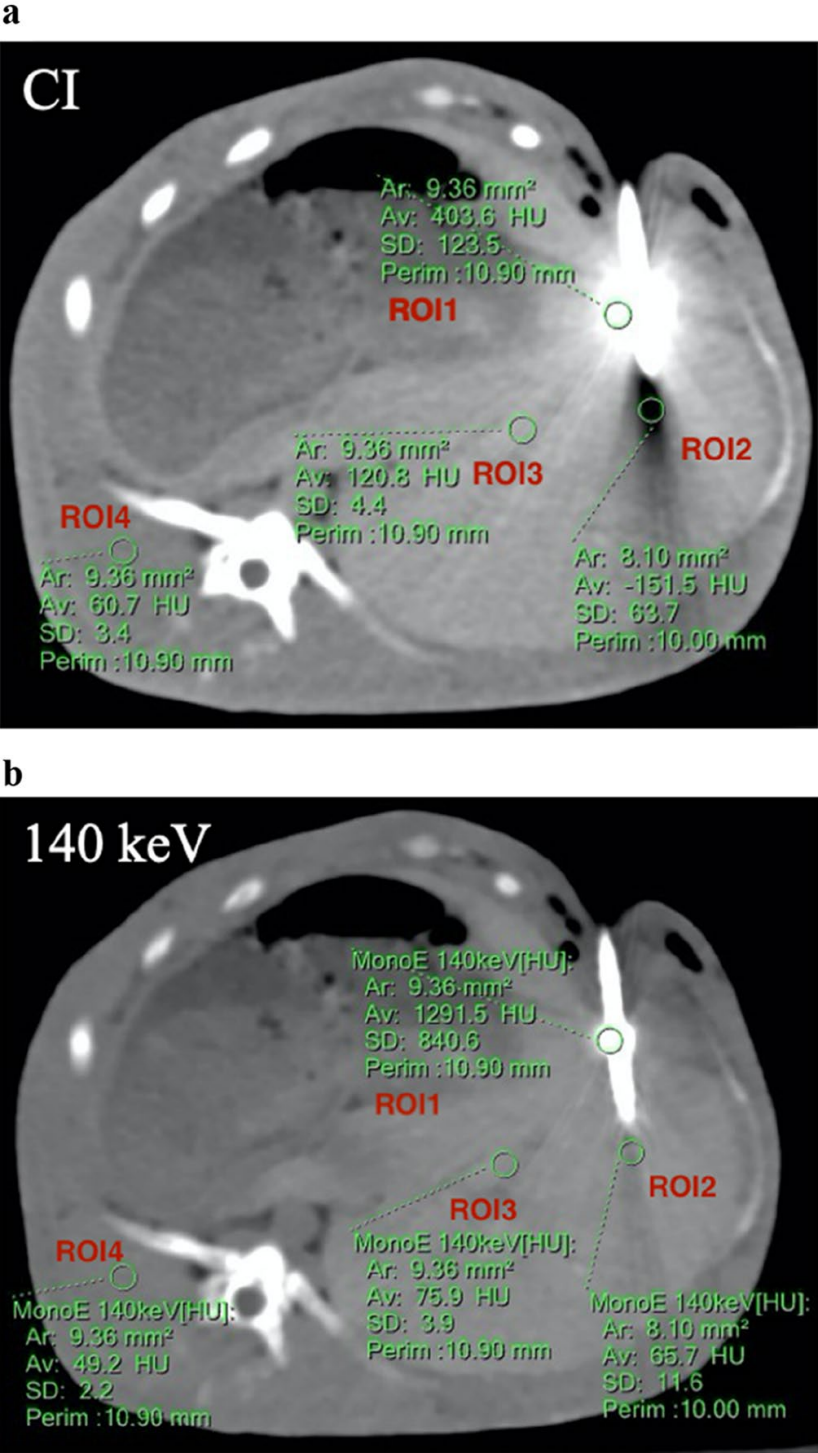
(**a**) Place the regions of interest on conventional images (CIs) in the hyperdense artifacts (ROI_1_), hypodense artifacts (ROI_2_), surrounding liver parenchyma with the presence of artifacts (ROI_3_) and distant regions of back muscles that were not affected by artifacts (ROI_4_). (**b**) The single virtual monoenergetic image with different levels was then “copy-paste” to ensure that the position, shape and size of the ROIs were exactly the same.

Objective image evaluation was performed by another radiologist with five years of experience in abdominal imaging. The elliptical regions of interest (ROIs) were placed in the following areas on CI first: hyperdense artifacts (ROI_1_), hypodense artifacts (ROI_2_), surrounding liver parenchyma with the presence of artifacts (ROI_3_) and distant regions of back muscles that were not affected by artifacts (ROI_4_). The CT attenuation values in Hounsfield units (CT_1_, CT_2_, CT_3_, and CT_4_) and standard deviations (SD_1_, SD_2_, SD_3_, and SD_4_) were recorded. Single VMIs with different levels were then “copy-pasted” in the same region to ensure that the position, shape and size of the ROIs remained the same. We ensured that the size of the ROI_1_ and ROI_2_ were completely within the range of hyperdense artifacts and hypodense artifacts, respectively. The size of the ROIs was approximately 15 mm^2^, which probably changed to adjust to the target lesion (Fig. 1a,b). We calculated the difference between the SD value in soft tissue impaired by artifacts (in the surrounding liver parenchyma) and the corresponding undamaged reference tissue (back muscle) as the corrected image noise (CIN) to eliminate the lower image noise in VMIs with high keV levels^10,12^. The corrected attenuation, which referred to the difference between the HU values in the region with (hyper- and hypodense artifacts, surrounding liver parenchyma) and without (back muscle) artifacts, was also calculated to be compared^12^.

### Statistical analysis

SPSS software (version 20.0, SPSS Inc., Chicago, USA) was used for statistical analysis. Discrete variables are expressed as the median and interquartile range, and continuous variables are described as the mean ± SD. The Shapiro–Wilk test was applied to test the normal distribution. The Friedman test was used for multiple comparisons between CI and VMI, including corrected attenuation of hyperdense artifacts, hypodense artifacts and surrounding liver parenchyma impaired by artifacts, corrected image noise and subjective scores. For variables with statistical significance, the Dunn-Bonferroni post-hoc test was performed to analyze all pairwise comparisons. The intraclass correlation coefficient (ICC) test was used for the consistency of observers in subjective evaluation of image quality under different levels of VMI: ICC > 0.75: excellent consistency, ICC > 0.6: good consistency, ICC > 0.4: moderate consistency, ICC ≤ 0.4: poor consistency^13^. P < 0.05 was considered statistically significant.

## Results

### Qualitative evaluation results

The subjective image quality scores determined by two radiologists showed great consistency (ICC = 0.925). The reduction of hyperdense artifacts was observed at 170–200 keV. The hypodense artifacts were decreased at 160–200 keV. The diagnostic evaluation of adjacent liver tissue was statistically higher in VMIs than CI, specifically at 140–200 keV.

New artifacts were presented in VMIs compared with CI reconstructions [VMIs at 90、100, 110, 120 and 140 keV: 2 out of 31 (6.5%); VMIs at 150 and 160 keV: 4 out of 31 (12.9%); VMIs at 170 keV: 8 out of 31 (25.8%); VMIs at 180 keV: 5 out of 31 (16.1%); VMIs at 190 and 200 keV: 7 out of 31 (22.6%)]; however, none of them impaired diagnostic image quality (Table 1).

**Table 1:** Qualitative assessment of artifact reduction and surrounding liver parenchyma.

	Artifact extent			New artifact
	Hyperdense	Hypodense	Surrounding liver parenchyma	
CI	1 (1,1)	1 (1,1)	2 (1,2)	–
40 keV	1 (1,1)	1 (1,1)	1 (1,1)	1 (1,1)
50 keV	1 (1,1)	1 (1,1)	1 (1,1)	1 (1,1)
60 keV	1 (1,1)	1 (1,1)	1 (1,1)	1 (1,1)
70 keV	1 (1,1)	1 (1,1)	2 (1,2)	3 (3,3)
80 keV	1 (1,2)	1 (1,1)	2 (2,2)	3 (3,3)
90 keV	2 (1,2)	2 (2,2)	2 (2,3)	3 (3,3)
100 keV	2 (2,2)	2 (2,2)	3 (3,4)	3 (3,3)
110 keV	2 (2,3)	3 (3,3)	3 (3,4)	3 (3,3)
120 keV	3 (2,3)	3 (3,3)	4 (3,4)	3 (3,3)
130 keV	3 (3,4)	3 (3,4)	4 (3,5)	3 (3,3)
140 keV	3 (3,4)	4 (3,4)	*5 (4,5)*	3 (3,3)
150 keV	4 (3,4)	4 (4,4)	*5 (4,5)*	3 (3,3)
160 keV	4 (4,4)	*4 (4,4)*	*5 (4,5)*	3 (3,3)
170 keV	*4 (4,5)*	*4 (4,5)*	*5 (5,5)*	3 (2,3)
180 keV	*4 (4,5)*	*4 (4,5)*	*5 (5,5)*	3 (3,3)
190 keV	*4 (4,5)*	*4 (4,5)*	*5 (5,5)*	3 (3,3)
200 keV	*5 (4,5)*	*5 (4,5)*	*5 (5,5)*	3 (3,3)
**P values**				
CI vs. 40–80 keV	P > 0.05	P > 0.05	P > 0.05	–
CI vs. 90–200 keV	P < 0.001	P < 0.001	P < 0.001	–

*CI* conventional images.

Data in italics indicate no statistical significance between any two energy levels.

### Quantitative evaluation results

**Figure 2:**
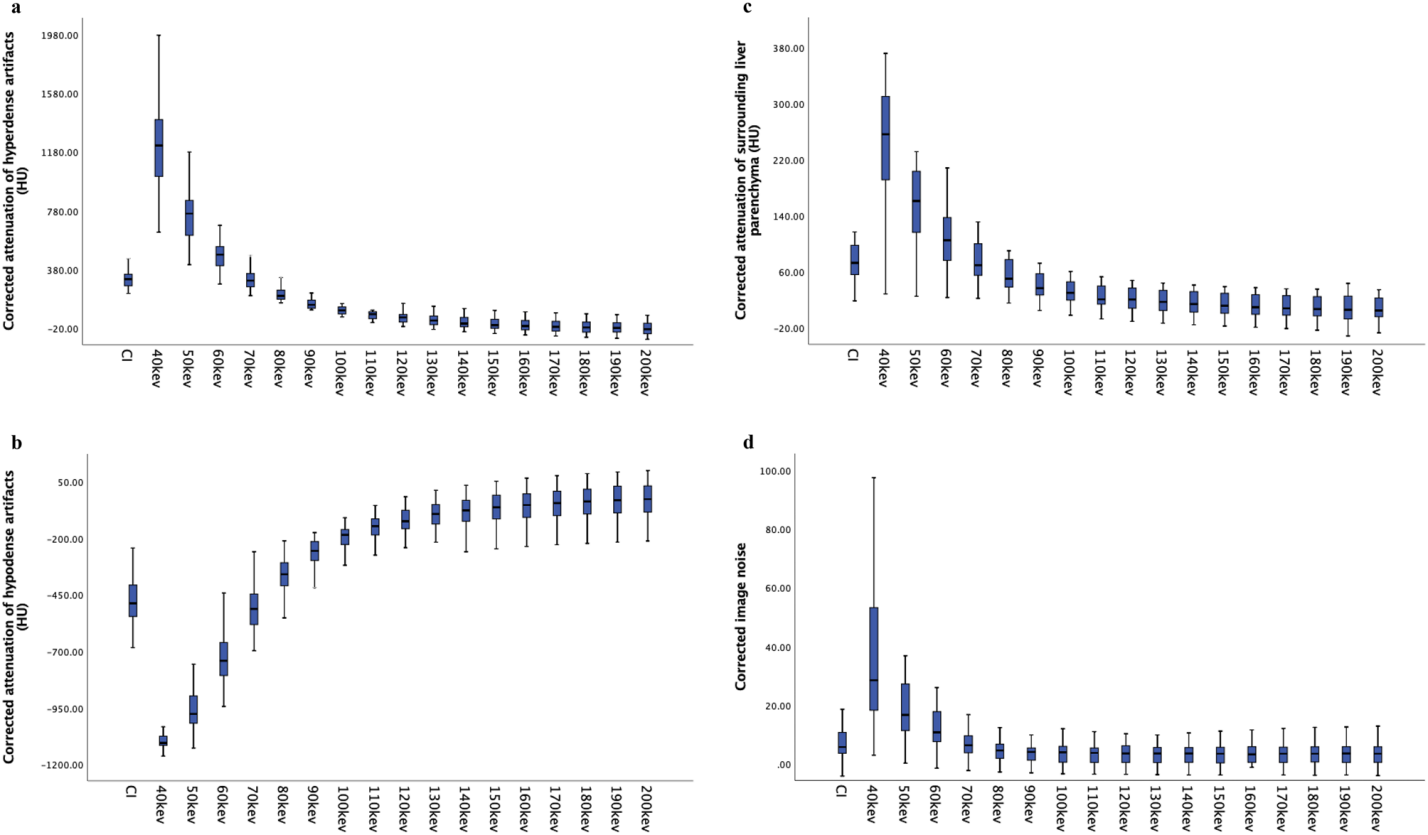
Box-plot diagram showing corrected attenuation of hyperdense artifacts (**a**), hypodense artifacts (**b**) and the surrounding liver parenchyma impaired by artifacts (**c**) and corrected image noise (**d**) on conventional images (CI) and virtual monoenergetic images (VMIs) at 40–200 keV. VMIs at high energy levels can significantly reduce corrected attenuation but cannot reduce corrected image noise compared to CI.

Compared with CIs, 90–200 keV reduced the corrected attenuation of hyperdense artifacts (P < 0.001), and the corrected attenuation decreased with the increasing energy levels. Notably, VMIs at 90–100 keV had a higher corrected attenuation value of hyperdense artifacts than those at 200 keV (P < 0.001). When the energy level was higher than 110 keV, the corrected attenuation of the strong hyperdense artifact was not significantly different between each energy level group. In contrast, 40–60 keV increased the corrected attenuation of hyperdense artifacts compared with CIs (P < 0.001). On the other hand, there was no significant difference between CIs and 70–80 keV considering the corrected attenuation of hyperdense artifacts (P > 0.05) (Table 2, Fig. 2a). Regarding the hypodense artifacts, VMIs at 40–60 keV allowed for extra increased hypodense artifacts compared with CIs (P < 0.001). The difference between 70 keV and CI was not statistically significant (P > 0.05). Higher energy levels at 80–200 keV enabled a decrease in hypodense artifacts compared with CIs (P < 0.001) and decreased extent with increasing energy levels. Notably, 80–110 keV had obviously more hypodense artifacts than 200 keV (P < 0.001), while 120 keV and even higher levels did not lead to an additional increase in hypodense artifacts (Table 2, Fig. 2b).

**Table 2:** Quantitative assessment of artifact reduction and surrounding liver parenchyma.

	Corrected attenuation			Corrected image noise
	Hyperdense artifact	Hypodense artifact	Surrounding liver parenchyma	
CI	337.9 ± 104.2	(−)479.2 ± 132.0	75.8 ± 25.5	7.0 ± 5.7
40 keV	1251.4 ± 380.4	(−)1074.6 ± 90.1	251.5 ± 101.6	34.1 ± 27.4
50 keV	772.7 ± 295.5	(−)928.5 ± 149.4	160.3 ± 62.8	19.4 ± 13.6
60 keV	508.9 ± 176.2	(−)711.3 ± 155.3	106.6 ± 40.7	18.4 ± 30.8
70 keV	330.1 ± 128.3	(−)516.4 ± 138.4	74.5 ± 28.5	8.1 ± 6.7
80 keV	230.4 ± 82.7	(−)378.2 ± 121.0	54.2 ± 22.2	5.6 ± 5.5
90 keV	160.4 ± 63.0	(−)284.1 ± 107.9	41.0 ± 19.1	4.5 ± 4.7
100 keV	113.0 ± 52.5	(−)218.8 ± 100.0	*32.3* ± *17.6*	4.0 ± 4.4
110 keV	*79.2* ± *47.9*	(−)173.2 ± 95.5	*25.7* ± *17.3*	3.8 ± 4.3
120 keV	*54.9* ± *46.2*	*(* −*)139.9* ± *93.5*	*21.1* ± *17.2*	5.0 ± 7.9
130 keV	*36.8* ± *46.0*	*(* −*)115.2* ± *92.7*	*17.7* ± *17.3*	3.8 ± 4.4
140 keV	*23.0* ± *46.5*	*(* −*)96.1* ± *92.4*	*15.1* ± *17.5*	3.9 ± 4.5
150 keV	*12.1* ± *47.3*	*(* −*)73.2* ± *105.9*	*13.0* ± *17.7*	3.9 ± 4.6
160 keV	*3.6* ± *48.1*	*(* −*)69.6* ± *92.7*	*11.5* ± *17.9*	4.2 ± 4.4
170 keV	*(-)3.3* ± *48.9*	*(* −*)60.1* ± *92.9*	*10.2* ± *18.0*	4.1 ± 4.7
180 keV	*(-)9.0* ± *49.7*	*(* −*)52.4* ± *93.2*	*9.1* ± *18.2*	4.1 ± 4.7
190 keV	*(-)12.2* ± *49.5*	*(* −*)44.6* ± *94.5*	*7.9* ± *20.5*	4.1 ± 4.7
200 keV	*(-)17.5* ± *51.0*	*(* −*)40.7* ± *93.7*	*7.5* ± *18.1*	4.1 ± 4.8
**P values**				
CI vs. 40–50 keV	P < 0.001	P < 0.001	P < 0.001	P < 0.001
CI vs. 60 keV	P < 0.001	P < 0.001	P > 0.05	P < 0.001
CI vs. 70 keV	P > 0.05	P > 0.05	P > 0.05	P > 0.05
CI vs. 80 keV	P > 0.05	P < 0.001	P > 0.05	P > 0.05
CI vs. 90–200 keV	P < 0.001	P < 0.001	P < 0.001	P > 0.05

*CI* conventional images.

Data in italics indicate no statistical significance between any two energy levels.

For the adjacent liver parenchyma impaired by metallic artifacts, its corrected attenuation value was statistically higher at 40–50 keV than CI (P < 0.001). There was no statistically significant difference between CI and 60–80 keV (P > 0.05). At 90–200 keV of VMIs, the corrected attenuation value of adjacent liver tissue decreased with the increasing energy levels. Within-group comparison showed that the value was the lowest when the energy level was 100–200 keV, and the difference was statistically significant (P < 0.001) (Table 2, Fig. 2c).

**Figure 3:**
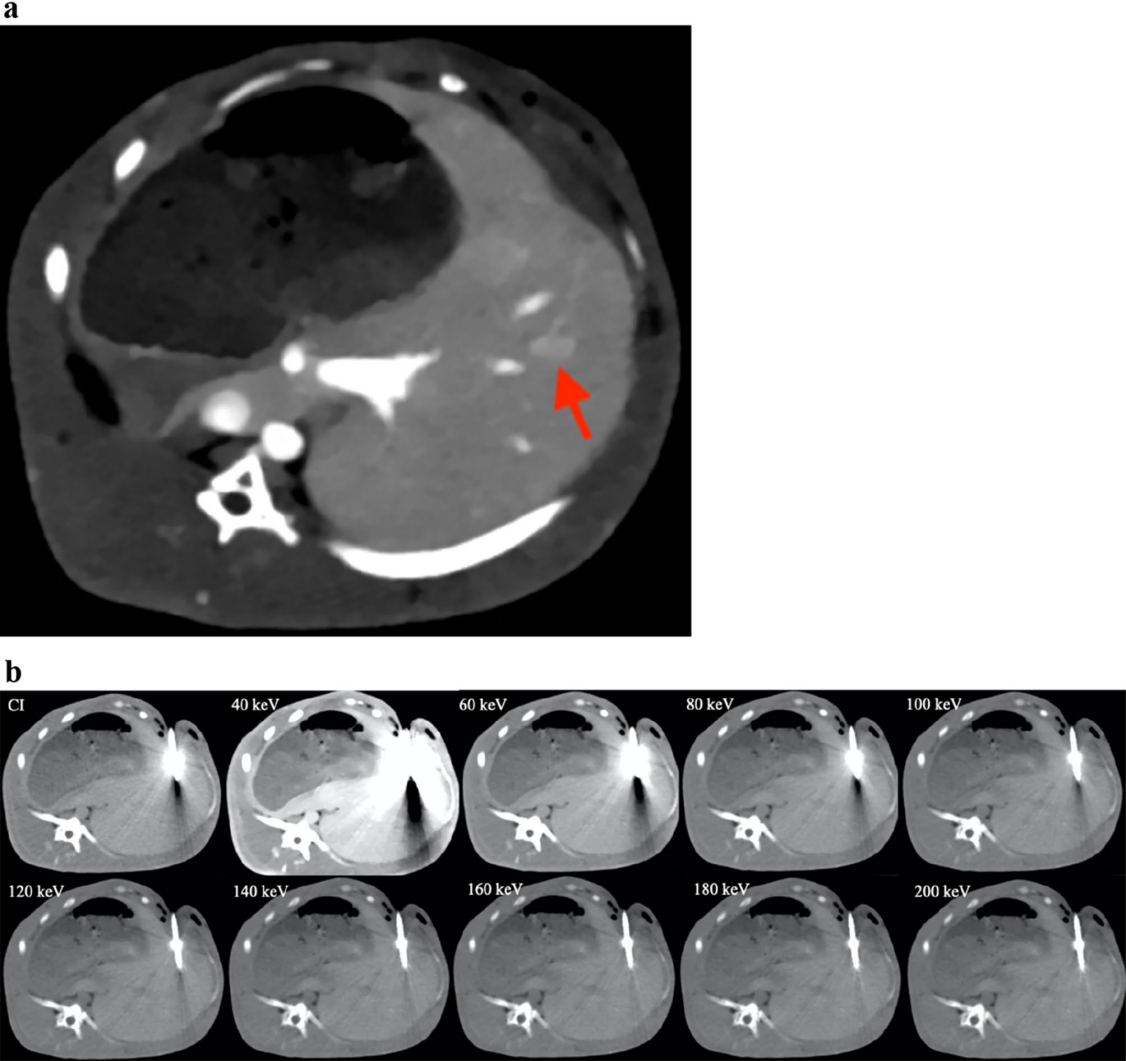
(**a**) Contrast-enhanced CT of VMIs at 40 keV showing the liver cancer (red arrow) in a rabbit. (**b**) Axial plain CT in the same rabbit with MWA treatment. Images were reconstructed as conventional images (CIs) and as different energy levels of virtual monoenergetic images (40 keV, 60 keV, 80 keV, 100 keV, 120 keV, 140 keV, 160 keV, 180 keV and 200 keV, window width/level: 350/60 for all images).

VMIs at 40–60 keV instead had a higher CIN than CIs (P < 0.001). However, as the energy level was further increased, no statistically significant difference was observed in CIN values between VMI and CI (P > 0.05) (Table 2, Figs. 2d, 3a,b).

### Radiation exposure

The CTDI_vol_ was 9.0 mGy for each rabbit.

## Discussion

This animal study objectively and subjectively evaluated the ability of VMIs to exhibit reduced metallic microwave ablation needle-related hyperdense or hypodense artifacts in a rabbit VX2 model. The results showed that VMIs at high keV could decrease the HU values of hyperdense artifacts and increase those of hypodense artifacts, which allowed the operator to clearly observe the location of the ablation needle and tumor lesion and provided a greater possibility for an accurate MWA procedure.

Traditional single-energy CT scanners adopt a higher tube voltage (kVp setting) or tube current (mAs setting) to reduce these artifacts, making it possible to bring more radiation dose to patients^14^. The appearance of VMIs generated from spectral CT provides another feasible method for MAR. The spectral CT system in the present study was equipped with a single source and a dual layer detector made of different scintillating crystal materials. When X-rays arrive through the detector, low-energy photons are absorbed in the top layer detector, and high-energy photons are absorbed in the bottom layer detector, allowing the separation of different energy levels of X-ray photons^15^. The data from these two detectors can be combined into a single projection data set, similar to the data from a conventional CT system. These data can be reconstructed by standard filtered projection or iterative reconstruction of mixed energy images. Moreover, the dual-layer detector can simultaneously receive and measure low-energy and high-energy X-rays projected from the same space and angular position for dual-energy postprocessing, with perfect time and space registration^16^. Therefore, dual-energy decomposition can be performed using the projection space, which makes the polychromatic CT image based on the dual-energy data processing method better than the traditional single-energy method, especially high energy level of keV in reducing the beam hardening correction related to metal implants^17^. In addition to the VMIs reconstruction series generated from this spectral CT system, other vendors also have their own techniques for VMIs reconstruction^18^, such as dual-source dual energy CT (SOMATOM Force, Siemens Healthcare, Erlangen, Germany)^19^ and rapid-kilovoltage-switching dual energy scanner (Discovery CT750 HD system with Gem-stone Spectral Imaging, GE Healthcare)^20,21^. Although the technical approaches are different, it has been suggested that the reconstruction of VMIs can make metal artifact reduction possible.

Several studies have suggested that VMIs is an efficient approach for artifact reduction. Wellenberg et al.^22^ found that VMIs led to an obvious reduction in metallic artifacts in a water-filled total hip arthroplasty phantom. A recent article^23^ evaluated the ability of VMIs to exhibited reduced hypo- and hyperdense artifacts generated from the port chamber and the distal tip of the port catheter. They hold that VMIs at high keV allows artifact reduction and then achieves improved image quality. Yoo et al.^24^ reviewed VMIs at 50–200 keV in 33 patients with metallic orthopedic implants. The authors concluded that 110–130 keV was the optimal energy range for showing the slightest artifacts and achieving the most satisfied image quality. Another study from Dangelmaier et al.^25^ also concluded that VMIs at high energy levels, especially at 180 keV, could significantly reduce the metallic artifacts of posterior spinal fusion. Our findings are similar to those depicted above. We found that VMI at a high keV level could reduce microwave ablation antenna-related hyper- and hypodense artifacts in a rabbit VX2 model, subjectively and objectively. This can improve the CT image quality around the ablation needle visually. In addition, VMI could also make the surrounding soft tissue impaired by artifacts more clearly revealed, which helped the accurate distribution of the ablation antenna. VMIs at each energy level can be reconstructed in real time with DLSCT, which has vital importance for the ablation process.

However, there was no statistical difference in image noise between CI and VMI at high energy levels in our research, which is consistent with a previous study^12^. Although VMIs can generate new artifacts as compared to CIs, these new artifacts do not affect the diagnostic assessment.

The following limitations need to be considered. First, we only had 31 rabbits in this study, and a larger sample size should be conducted to verify the outcomes. Second, the experiment was performed in a rabbit model. Although CT-guided MWA is also feasible in human patients. It may be limited to spreading our findings currently in clinical practice given the congenital difference between the rabbit VX2 model and human liver cancer. Nonetheless, we believe that this study provides reliable preclinical evidence that can significantly reduce the impact of ablation needle-related metallic artifacts and further improve ablation efficiency. Future prospective clinical studies are necessary to evaluate its application in human patients. In addition, the survival and outcomes of rabbits are ignored since this experimental study mainly focused on the image quality of VMIs. This should be further supplemented and improved in future research. Due to the special time requirements of the ablation process, although VMI can allow convenient and complete reconstruction, it does require additional reconstruction time. In practical clinical applications, the reconstruction algorithms may need to be further improved.

## Conclusion

In conclusion, VMI can obviously reduce artifact interference and provide better image quality visually during the CT-guided MWA process in a rabbit VX2 model. VMI at 170–200 keV is the best observation energy level for decreasing metal artifacts around the ablation zone, reducing image noise, ensuring image contrast, and improving subjective image quality; these characteristics are advantageous for suitable visualization of the ablation needle and surrounding tissue structure, as well as the relationship between the location of the lesion. VMIs makes it possible to acquire clearer image series, which in turn provides theoretical support for future clinical practices.
